# Treatment of Carbuncle

**Published:** 1877-08-20

**Authors:** C. B. Leitner

**Affiliations:** Columbus, Ga.


### TREATMENT OF CARBUNCLE.

BY C. B. LEITNER, M.D., COLUMBUS.

Having been called upon to treat three or four carbuncles at intervals of four to six months, I adopted the following course, with the best results:

It is known to the profession that generally before medical aid is called in, the usual routine of domestic remedies has been exhausted. The plan which I have adopted has been to apply a large cupping glass, and exhausting until all the matter in the cribriform cells has been drawn into the glass, then remove it, and wash the place with a solution of carbolic acid; one part of acid to eight of water—keeping a cloth saturated with this solution on the carbuncle. When it becomes painful again (this having relieved it for a while), re-apply the glass. From two to three times will exhaust the matter.

A very moderate drawing of the cup should be kept up until all the matter is removed. Upon the first application the patient will complain, but after that, as soon as he experiences the peculiar throbbing pain, he will call for the cup again. Another advantage that accrues from this process, is the compression of the large blood-vessels which supply the ulcerated cells.

I would advise at every application, that the cups he placed in the same indenture. for thus they act as compresses upon the large veins which have become dilated by the inflammation.

I could report cases that have terminated very favorably from this course of treatment in from three to four days. A gentleman aged about fifty years, had a ca buncle a little to the right of the spine, over the upper portion of the right scapula , he had not slept for forty-eight hours. I applied a large glass with a piece of pape saturated in alcohol, which removed about four ounces of sanguineous puss, dr s ed the wound with carbolic dressing. and in less than half an hour, he was asleep, and slept four hours and a half without a narcotic.

With my experience in this treatment, I cannot too highly recommend it to the professicn.

It is new to me, but may be old to some others; yet none of the authors whom I have consulted make the slightest allusion to it.

I have employed the same method in the exhaustion of boils and bone felons. After opening, I apply a common glass syringe, removing the rod and piston and placing the nozzle over the orifice, I draw the breath through the smaller end. This process has accomplished more in one application than poultices would do in three days. Should I be called to see a case of carbuncle at its formation, when in the stage of a hard, circumscribed, livid-red swelling, and with severe burning and smarting pains, which are always present in the first stages, I would set the blades of the scarificator to cut deeply into the part, and then turn and cross each incision with the same instrument, then apply the cupping glass, and I have not a doubt but that it would prevent the formation of the carbuncle, particularly if the cut was applied two or three times.

The internal remedies which I have found best, are iodide of potassum, quinine and iron.—*Trans. Med. Association of Georgia.*

